# Flexibility Is Costly: Hidden Physiological Damage From Seasonal Phenotypic Transitions in Heterothermic Species

**DOI:** 10.3389/fphys.2020.00985

**Published:** 2020-08-12

**Authors:** Julie Landes, Samuel Pavard, Pierre-Yves Henry, Jérémy Terrien

**Affiliations:** ^1^Unité Mécanismes Adaptatifs et Evolution (MECADEV), UMR 7179, CNRS, Muséum National d’Histoire Naturelle, Brunoy, France; ^2^Unité Eco-anthropologie (EA), Muséum National d’Histoire Naturelle, CNRS, Université de Paris, Paris, France

**Keywords:** flexibility, seasonal transitions, heterothermy, hypometabolism, physiological costs, aging

## Abstract

Heterothermy allows organisms to cope with fluctuating environmental conditions. The use of regulated hypometabolism allows seasonal heterothermic species to cope with annual resource shortages and thus to maximize survival during the unfavorable season. This comes with deep physiological remodeling at each seasonal transition to allow the organism to adjust to the changing environment. In the wild, this adaptation is highly beneficial and largely overcomes potential costs. However, researchers recently proposed that it might also generate both ecological and physiological costs for the organism. Here, we propose new perspectives to be considered when analyzing adaptation to seasonality, in particular considering these costs. We propose a list of putative costs, including DNA damage, inflammatory response to fat load, brain and cognitive defects, digestive malfunction and immunodeficiency, that should receive more attention in future research on physiological seasonality. These costs may only be marginal at each transition event but accumulate over time and therefore emerge with age. In this context, studies in captivity, where we have access to aging individuals with limited extrinsic mortality (e.g., predation), could be highly valuable to experimentally assess the costs of physiological flexibility. Finally, we offer new perspectives, which should be included in demographic models, on how the adaptive value of physiological flexibility could be altered in the future in the context of global warming.

## Introduction

In seasonal environments, resource availability fluctuates over the year, mainly between a phase of resource abundance and a phase of resource shortage. Heterothermy is an adaptation to these seasonal changes, which are mainly periodic and therefore predictable, in environmental conditions. Heterothermy is defined as “the pattern of temperature regulation” in an endothermic species (i.e., mammal or bird) “in which the variation in core temperature, either nychthemerally or seasonally, exceeds that which defines homeothermy” [IUPS Thermal Commission (The Commission for Thermal Physiology of the International Union of Physiological Sciences), 2003]. To cope with seasonal fluctuations, heterothermic species show great variations in their metabolic activity. They alternate between an active/reproductive state with a high metabolic rate during the favorable phase and an inactive/resilient state with a low metabolic rate and reduced responsiveness to stimuli [i.e., state of torpor; IUPS Thermal Commission (The Commission for Thermal Physiology of the International Union of Physiological Sciences), 2003] during the unfavorable phase (Gwinner, 1986; Ruf et al., 2012; Ruf and Geiser, 2015). In this article, we chose to focus exclusively on seasonal heterotherms (hereafter abbreviated as “SH”) ‘that employ hypometabolism […] on a seasonal basis’ (Lyman et al., 1982; Ruf and Geiser, 2015), as opposed to opportunistic heterotherms that use hypometabolism as an emergency life-history stage (Wingfield, 2003; Nowack and Dausmann, 2015). There is a difference between obligatory seasonal remodeling (predictable; synchronized with the photoperiod) and unpredictable environment-dependent adjustment (corresponding to the plasticity of opportunistic expression of heterothermy). SH use seasonal changes in the photoperiod as an environmental cue to synchronize their physiology to the environment and to adjust their metabolic rate to undergo either hibernation (i.e., with torpor episodes lasting weeks) or daily torpor during unfavorable phases (Geiser, 2017). Photoperiod-induced changes in metabolic activity (and reproduction) are so deeply imprinted in these species’ genomes that we still observe them in captivity where resources are constantly abundant, even after many generations (e.g., Perret et al., 1998). This predominant role of the photoperiod does not preclude the roles of other environmental cues, e.g., food availability, in the expression patterns of heterothermy (Vuarin et al., 2015). SH show specific changes in several physiological and behavioral functions between seasons. For instance, torpor use is linked with the alteration of cell membrane function, neural and cardiovascular functions, skeletal muscle function, immune function, anorexia and nonshivering thermogenesis, or lipidic metabolism and fatty acid saturation (Carey et al., 2003; Klug and Brigham, 2015).

**FIGURE 1:**
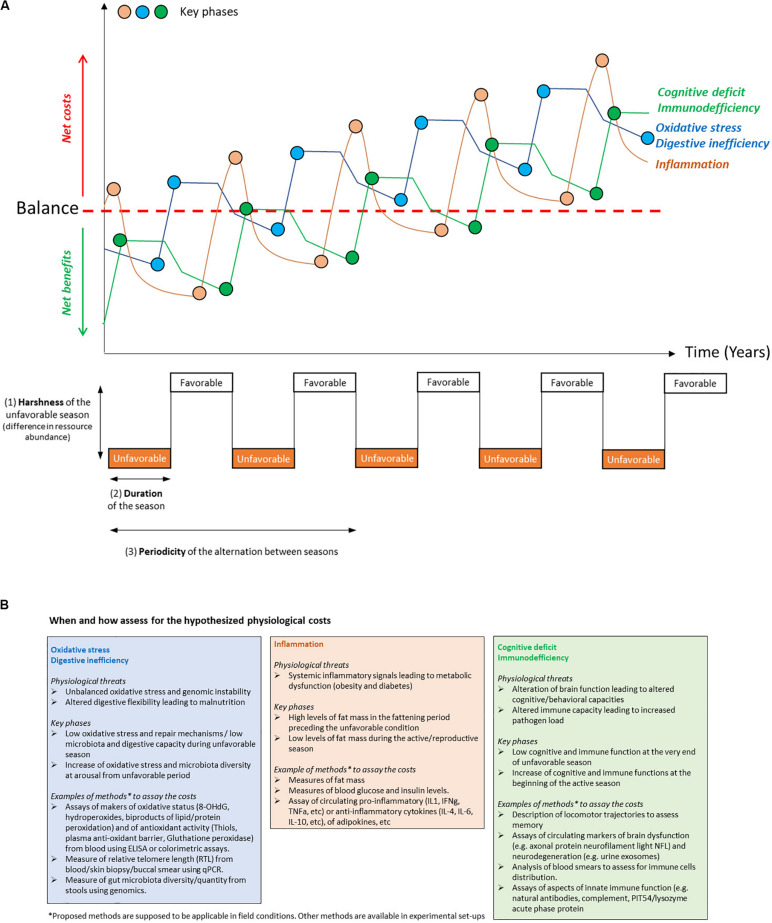
(**A**-Top panel) schematic representation of the hypothetical changes over time in the cumulative damage induced by different physiological costs of heterothermy and the corresponding key phases when these costs are likely to occur. These changes account for phases of bursts and phases of uncomplete restoration of the considered damages. The red dotted line represents the threshold at which the balance between the benefits and the costs for each considered physiological feature is null. Each cost emerges early in life but is only partially compensated for, therefore leading to the accumulation of damage with age. (**A**-Bottom panel) parameters, i.e., harshness (1), duration (2), and periodicity (3), involved in the seasonal transitions undergone by heterothermic species. **(B)** Examples of physiological costs of heterothermy, the key time phases at which they may occur, and proposed methods to assay these costs.

Heterothermy comes with both ecological and physiological benefits (Nowack et al., 2017). Overall, these benefits have to largely exceed the potential costs in fluctuating environments (Lovegrove, 2012; Lovegrove et al., 2014). However, these costs have been largely neglected by researchers. Yet it is important to consider these costs for several reasons. First, understanding how physiological transitions affect both benefits and costs in survival and reproduction and when they occur throughout life is crucial to assess the effects of environmental fluctuations (in harshness, duration and periodicity, see Figure 1A) on individual fitness. Second, understanding the constraints linked to heterothermic life cycles (i.e., the covariance between life-history traits resulting from the interactions between environmental conditions and physiological transitions) will allow us to better integrate heterothermic strategies within life-history theory.

In this paper, we propose new perspectives on the marginal costs of seasonal flexibility. We do not intend to provide an extensive review of the literature on the regulation of seasonal heterothermy but rather to shed light on putative physiological costs that have been overlooked so far and should be considered in future research. We propose that these costs should be better supported by experimental evidence to be further included in studies modeling the interactions between heterothermy and environmental changes (Boyles et al., 2020).

## The Obvious Ecological Benefits of Seasonal Heterothermy… and Its Costs

Heterothermy brings ecological benefits. First, matching metabolic activity with resource availability maximizes survival, as the organism is less constrained by energy and water shortages. The match of metabolic activity to resource fluctuations in SH also enhances reproductive success. Second, heterothermy promotes metabolic flexibility, i.e., the ability to adjust the origin of substrates used for oxidative metabolism, switching from carbohydrate to lipid usage. Metabolic flexibility is thought to enhance the control of energy balance and has even been proposed to favor longevity (Goodpaster and Sparks, 2017; Smith et al., 2018). Regulated hypometabolism (as opposed to unregulated, pathological hypothermia; Barros et al., 2001) has been shown to increase survival and to lower the senescence rate during the unfavorable season (e.g., Liow et al., 2009; Turbill et al., 2011, 2012). It also often correlates with reproductive inactivity (e.g., Canale et al., 2012), which, according to the disposable soma theory of aging (Kirkwood and Holliday, 1979), could also promote repair mechanisms and slow down the senescence process, as energy is not invested in reproduction (Ricklefs and Wikelski, 2002).

However, seasonal heterothermy may also come with ecological costs. In the wild, these costs are compensated by the benefits of heterothermy and are therefore hidden (not observable). However, several recent studies revealed such costs. For example, some species avoid using torpor when environmental conditions are good enough (e.g., Landry-Cuerrier et al., 2008; Levy et al., 2011). This suggests that the benefits of using torpor only hold in unfavorable conditions and that expression of torpor when not necessary might come with costs. Organisms using heterothermy are also shown to perform poorly in terms of resource acquisition because of a phenological trophic mismatch. For example, mice using torpor emerge later than nontorpid mice and “miss” the period of good resource availability (Levy et al., 2012). When they emerge, competition for food is high, and the best resources are no longer available. Therefore, mice using torpor show less energy intake and compensate by using longer torpor periods, which contribute to maintaining the mismatch. Another potential ecological cost of heterothermy is increased predation risk during torpor and at the beginning of the active period due to decreased locomotor ability and reduced perception of the environment (Humphries et al., 2003; Carr and Lima, 2013), although torpor use also reduces exposure to predators (e.g., Turbill and Stojanovski, 2018). Torpor also leads to dehydration, which implies compromised circulation and modification of the ionic balance (Humphries et al., 2003). The decrease in locomotor abilities associated with torpor use also leads to an increase in hoard pilferage risk for food-storing SH (Humphries et al., 2003).

## Physiological Costs of Heterothermy Emerge Early in Life and Accumulate With Age

**FIGURE 2:**
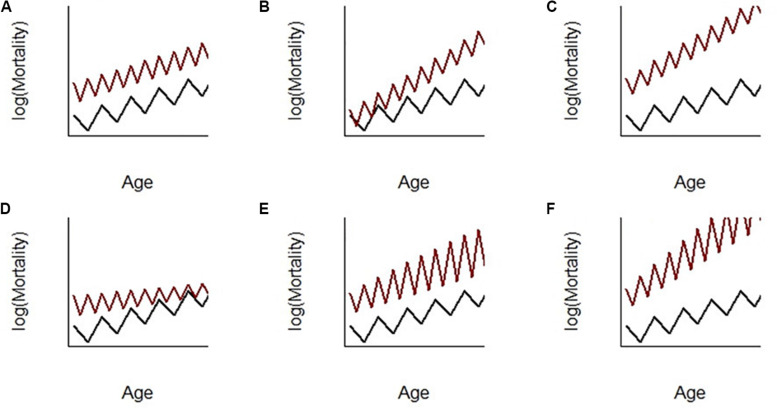
Scenarios for the potential effect of seasonal transitions (in frequency and magnitude) on mortality patterns with age. Age-specific mortality *h(t)* is modeled by a Gompertz-shaped function such that the mortality hazard at age *t* is *h*(*t*) = (*ae*^*sr*.α^)(*e*^*bte*^*sr*.β^^)(*e*^*s*.(γ*t*+σ)^), where (i) the first term is the proportional hazard (PH) component of the Gompertz parameter *a*, with *sr* (for seasonal rhythm) being the covariate describing the frequency of the alternation between seasons and α being its PH effect; (ii) the second term is the accelerated failure time (AFT) component of the Gompertz parameter *b*, with β being the AFT effect of variable *sr*; (iii) the third term is the effect of the Boolean variable season *s* on mortality, with a magnitude σ and a potential increase or decrease of the magnitude of fluctuations with age *t* according to the sign of γ. **(A)** PH effect of *sr* on mortality; **(B)** positive AFT effect of *sr* on mortality; **(C)** combined PH and positive AFT effect; **(D)** PH and negative AFT effects with a decreased magnitude of fluctuations with age; **(E)** PH effect with an increased magnitude of fluctuations with age; **(F)** PH and positive AFT effects with an increased magnitude of fluctuations with age.

There is an apparent paradox between the fact that seasonal heterothermy is an adaptation to predictable environmental changes and the recent hypothesis that the associated circannual physiological changes are detrimental to the organism in terms of increased mortality and early emergence of aging phenotypes (Landes et al., 2017). Indeed, the seasonal transitions between inactive and active metabolic states, as manifestations of phenotypic plasticity, come with a major remodeling of organismal physiology. For example, the changes in metabolic rate and body temperature are due to finely tuned modifications, such as changes in the expression of enzymes involved in anabolic and catabolic metabolism, erythropoiesis, fuel sources, regulation of protein transcription, membrane composition and thermogenesis (Ramenofsky and Wingfield, 2007; Klug and Brigham, 2015; Ruf and Geiser, 2015). This remodeling comes with generic costs of phenotypic plasticity referred to as maintenance, production, information acquisition, developmental instability and genetic costs (reviewed by Auld et al., 2010). Such costs may appear early in life, at key phases where they could be measured (Figure 1) and may affect mortality (Box 1 and Figure 2A; Landes et al., 2017).

**Box 1. Potential demographic effects of seasonal transitions on aging.**

We discuss here several possible ways in which frequencies and magnitudes of seasonal physiological transitions may shape mortality over age.

Figure 2A depicts the case where doubling the frequency of transition each year has a proportional hazard effect (PH) on mortality. This was observed in female gray mouse lemurs (*Microcebus murinus*) by Landes et al. (2017). In this case, the mortality of individuals subjected to accelerated seasonal transitions was multiplied by a factor independent of age (i.e., a relative risk). As a consequence, mortality rose faster in accelerated individuals (the slope *h’(t)* of mortality at a given age was increased). This supports accelerated aging in line with empirical observation that accelerated individuals exhibit early appearance of phenotypes and pathologies associated with aging (Aujard et al., 2001; Cayetanot et al., 2005). However, in this case, the rate of aging *h’(t)/h(t)* (i.e., how much mortality increased with age with respect to the level of mortality at this age) was constant.

An alternative effect of seasonal transitions could have been an accelerated failure time effect (AFT, Figure 2B). In this case, the frequency of transition changed how the time unit affected mortality and changed the parameter *b* of the Gompertz function. For instance, 1 year of time of reference individuals was equivalent to 2 years for accelerated individuals in Figure 2B and, consequently, accelerated individuals senesced twice as fast as reference individuals. Accelerated individuals clearly aged faster here since both *h’(t)* and *h’(t)/h(t)* were larger in accelerated individuals. This model would have been a perfect fit for the expectation that increasing transition frequency accelerates biological aging. This was, however, not supported by the data in Landes et al. (2017), even when testing for a combination of both PH and AFT effects (as in Figure 2C) following the statistical formulations in Landes et al. (2017), Supplementary Material, Appendix 2). Although such an AFT effect was not evidenced in Landes et al. (2017), this hypothesis still seems probable and should be further explored in future research.

Let us now focus on changes in the magnitude of mortality fluctuations with age. Figure 2D depicts the case where the magnitude of mortality fluctuations declines with age in accelerated individuals. This was observed in male gray mouse lemurs (alternative PH-(negative)-AFT model in Landes et al., 2017, supplementary table 2). Here, young adult accelerated males had, as did females, a proportional increase in mortality with age, but mortality increases fast in the young ages and then more and more slowly while individuals age (β < 0). It seems therefore that because males’ seasonal transitions had a lower amplitude with age (illustrated by reduced body mass fluctuations), the cost in terms of mortality also decreased with age. This is interesting because, if proven true, this would be a case where deterioration of the ability to perform seasonal transitions with age leads to a decreasing aging rate. To better emphasize the originality of these results, we provided in Figures 2E,F two alternative models that should fit the data if senescence of physiological transitions leads to an increase in the magnitude of mortality fluctuations without (Figure 2E) or with (Figure 2F) a positive AFT effect on the aging rate (although the models did not fit the data in Landes et al., 2017).

### Experimental Evidence of the Costs of Seasonal Physiological Remodeling

Increased frequency of seasonal transitions has been linked to increased mortality in captive gray mouse lemurs (*Microcebus murinus*; Landes et al., 2017). In this study, individuals were exposed to different numbers of seasonal transitions per year. Those experiencing more frequent seasonal transitions than usual showed an increase in mortality that was already visible at a very young age, therefore potentially contributing to faster aging (see Box 1). Functionally, increasing the frequency of seasonal transitions is known to lead to disorders, such as the emergence of altered circadian rhythmicity similar to the ones exhibited by aged individuals under a natural seasonal rhythm (Cayetanot et al., 2005). Interestingly, the animals that were put on accelerated seasonal rhythms (up to five seasons per year instead of two) were all properly “seasonally” entrained in terms of many of their physiological features (e.g., body mass, body temperature, reproduction, brain function) and did not show any sign of discomfort or illness. However, further increases in the frequency of seasonal transitions (i.e., more than five transition events per year) clearly showed that animals were not responsive to the change in photoperiod and escaped to the imposed seasonal rhythms as a manifestation of photorefractoriness (unpublished data; Martine Perret, personal communication). This demonstrates that the remodeling that occurs at each transition requires time and that there is a limit to the number of times that the organism can adjust. In addition, an increased frequency of seasonal transitions may increase the associated costs, as animals showed a more rapid decline with age in melatonin production and in their suprachiasmatic response to light (Aujard et al., 2001).

### Repair Mechanisms Are Altered During Aging

Extreme physiological performance (“profound physiological remodeling” in Regan et al., 2019) jointly evolved with specific physiological compensatory mechanisms that protect from and/or restore inevitable somatic damage. For instance, if an extreme metabolic performance generates above-normal oxidative stress, it usually comes along with the overexpression of enzymes that protect against/or repair DNA and cell membrane damage (Figure 1A). Aging results from the accumulation of damage to the organism over the lifetime, for which repair mechanisms no longer fully and efficiently compensate. Indeed, damage accumulates during each season, but repair processes (e.g., antioxidant enzymes in the case of oxidative stress) compensate, at least partially, for this damage to restore proper function at the adult stage (Figure 1A). In this case, the net benefits of heterothermy fully balance the costs, which are, therefore, not necessarily observable. Because the ability to perform this damage compensation decreases with age, for example, at the mitochondrial (Babbar et al., 2020) or genomic (Petr et al., 2020) levels, and because of the progressive accumulation of this damage, seasonal organisms may not suffer from the cost of physiological transitions before advanced age. Therefore, as compensation is not fully complete at each seasonal transition (Figure 1A), damage emerges early and further accumulates over age to reach a limit where the net costs overcome the benefits (Figure 1A). This principle of balance between damage and protective/repair responses applies to the different hypothetical physiological cost mechanisms listed hereafter. However, the cost of transitions would not be easily observed in the wild, where old ages are not often reached. Moreover, the ecological and physiological benefits of torpor expression hide these potential costs in the wild, a feature that does not hold in captivity where environmental constraints are mild. However, the study of individuals in captivity, where extrinsic mortality is negligible and more individuals reach advanced ages, promotes the observation of such costs. Captivity also allows control of photoperiodic entrainment and therefore facilitates access to animals reaching key phases when costs may arise and thus facilitates their assessment (Figure 1). Moreover, the links between seasonal transitions and aging are not trivial and need further investigation (see Box 1).

### Heterothermy Affects the Aging Process

In SH, such as the gray mouse lemur, the mortality pattern over age is driven by seasonality. The link between seasonal transitions and aging is interesting, as aging can be interpreted as the manifestation of damage accumulated at multiple levels. Indeed, seasonal physiological transitions must affect one or several biological functions, therefore contributing to the senescence process, which becomes apparent with the assessment of biological markers of aging (López-Otín et al., 2013). Some of these markers directly show the cause of the accumulating cellular damage, such as genomic instability, telomere attrition or epigenetic alterations. Other markers are the response of the organism to accumulating damage that becomes deleterious after a certain age, such as the loss of proteostasis, deregulation of nutrient sensing, mitochondrial dysfunction or cellular senescence.

Experimentally, regulated hypometabolism can be triggered in different ways, including food shortage or caloric restriction, thus mimicking phases of food scarcity in wild conditions (Vuarin and Henry, 2014). In this context, caloric restriction experiments have also evidenced hidden costs of regulated hypometabolism. These experiments have been extensively studied to manipulate intrinsic functioning and the pace of senescence in a large variety of species, including primates (Mattison et al., 2017; Pifferi et al., 2018). These studies showed that organisms under caloric restriction exhibit a decrease in metabolic rate and energy expenditure (DeLany et al., 1999; Redman et al., 2018), an enhanced lifespan and a delayed emergence of biomarkers of aging and age-related phenotypes (Mattison et al., 2017; Pifferi et al., 2018). However, these studies did not yet provide evidence on the extent to which metabolic depression following caloric restriction mimics a reduction in the pace of life and/or influences the speed at which mortality increases with age. In contrast, other studies suggest that increased mitochondrial uncoupling may also decrease oxidative stress and increase longevity (e.g., Caldeira da Silva et al., 2008). However, different studies support the hypothesis of an energy trade-off between somatic maintenance and reproduction during caloric restriction (e.g., Sitzmann et al., 2008, 2010; Pifferi et al., 2018), which may also hold during seasonal hypometabolic episodes. This is further supported by empirical data showing a negative correlation between the use of regulated hypometabolism and reproductive success in hibernating eastern chipmunks (Dammhahn et al., 2017).

Thus, adaptation to seasonal environments comes at a cost, which might translate into increased mortality risk. However, the underlying mechanisms linking the costs of seasonality and survival remain misunderstood. Here, we provide a list of putative physiological costs that should be further considered in experimental work to determine whether these costs are real and could contribute, to some extent, to minimizing the benefits of heterothermy and further accelerating the rate of aging.

## Putative Physiological Costs of Heterothermy

We expect physiological costs of seasonal heterothermy to emerge from imperfect biological regulations, imperfect seasonal regulation of competing functions and from suboptimal phenotypic matching with environmental changes. This recurrent phenotypic remodeling contributes to the accumulation of damage and thus to the aging process in SH (see Figure 1A). Although experimental evidence on each of these costs is very weak, the associated deep physiological remodeling has to be costly (Auld et al., 2010). Here, we will discuss five types of potential physiological costs of heterothermy that seem especially potent and relevant according to our knowledge on seasonality in SH. Indeed, in addition to their relevance from ecological perspectives, these five physiological costs are hot topics in other disciplines, including biomedical research (e.g., anti-inflammatory and antioxidant mechanisms), which would be interesting to take into account when studying heterothermy. For each cost, Figure 1 proposes an example of the potential evolution with time of the associated damages (Figure 1A), including phases of bursts and of incomplete restoration of damages and the key time phases at which these costs may occur (Figures 1A,B). We also provide some methodological examples that could be used to assay these costs in captivity and that are applicable to the field (Figure 1B).

### Oxidative Stress and Genomic Instability

As mentioned earlier, biological aging is mechanistically linked with metabolism since cellular functioning generates free radicals that cause cellular damage (Selman et al., 2012). The excess of free radicals compared to the cellular antioxidant response leads to oxidative stress. Under such circumstances, organisms experience a loss of functionality and a loss of cellular control, linked, for example, with cancer emergence when cells develop resistance capacities to high levels of oxidative stress (Sosa et al., 2013). Thus, the effect of oxidative stress on senescence depends on the balance between damage and molecular/cellular repair efficiency. As a consequence, the observed oxidative stress can be either directly the cause of DNA damage accumulation with age (i.e., genomic instability, telomere attrition, or epigenetic alterations), the responses to the damages becoming deleterious with age through impaired cellular processes (i.e., loss of proteostasis, deregulated nutrient sensing, mitochondrial dysfunction, or cellular senescence), or phenotypic results of the two previous phenomena leading to functional decline (i.e., stem cell exhaustion and altered intercellular communications; López-Otín et al., 2013). In SH, regulated hypometabolism is linked with low oxidative stress, such as in Arctic ground squirrels (Orr et al., 2009). In addition, variations in relative telomere length (a marker of aging) in SH model species show an elongation of telomeres during the hypometabolic state (Turbill et al., 2013). Telomere attrition is a marker of biological aging and apparently compromises DNA integrity; oxidative stress is thus low during regulated hypometabolism. However, arousal from a hypometabolic state may generate a brief but massive peak in oxidative damage (Figure 1). In mouse lemurs, when metabolic activity increases, DNA oxidative damage increases and relative telomere length decreases (Terrien et al., 2017). Telomere shortening during this seasonal transition may be an adaptive response. Indeed, this attrition would contribute to the amplification of the signaling of metabolic debt and therefore to the prioritization of somatic maintenance processes (Casagrande and Hau, 2019). In this respect, telomerase, whose activity varies across species, cell types and life stages (Gomes et al., 2010), represents the most widespread repair mechanism in normal cells. Interestingly, while telomerase activity in somatic tissues has been lost in some species, including humans, there is experimental evidence of maintained telomere repair capacity in normal somatic cells in SH (Wang et al., 2011; Gorbunova et al., 2014; Trochet et al., 2015). One could therefore hypothesize that maintenance of telomerase activity in somatic tissues is one feature of SH and that this repair mechanism might concur with extended longevity compared to that of homeotherm species (Turbill et al., 2011). However, although major focus has been placed on the potential of telomerase reactivation in anti-aging strategies, there exists little evidence that telomerase activity is impaired with age and that this translates into altered repair capacity (Anchelin et al., 2011).

### Excess Fat and the Associated Inflammatory Response

Another potential physiological cost of heterothermy is the risk associated with massive variations in body condition and, more particularly, fat reserves (Figure 1). For instance, SH often anticipate the harsh season by experiencing massive fattening (+50 to +100%) to build energy reserves on which they will rely for weeks or months. What remains exceptional, and not fully understood, is their faculty to store massive amounts of fat with no adverse effects. Briefly, excess fat, inducing metabolic imbalance, usually induces chronic low-grade systemic inflammation (Lumeng and Saltiel, 2011), leading to increasing circulating levels of proinflammatory and neurotoxic mediators. These signals migrate to the brain to trigger neuroinflammation, preceding the onset of obesity and insulin resistance (Thaler et al., 2012). In SH, a paradox is emphasized, as insulin sensitivity seems to be preserved during the obesogenic phase, which could be mediated by altered adipose PTEN/AKT signaling (Rigano et al., 2017). Recent work on the mouse lemur has shown that the relative expression of phospho-IRS-1 was enhanced in muscle during torpor but decreased in white adipose tissue, thus suggesting an inhibition of insulin/IGF-1 signaling during torpor in these tissues (Tessier et al., 2015). In parallel, animals seem to be protected from an inflammatory response during massive fattening (Terrien et al., 2017). Further work is needed to determine to what extent the mechanisms that prevent insulin resistance and inflammation during fattening are altered with age.

### Cognitive and Brain Dysfunction

During regulated hypometabolism, locomotor and sensory capacities are decreased temporarily (Figure 1), which is linked with reversible cerebral disorders (tau phosphorylation, loss of neuron functionality) and sleep debt (impaired memory consolidation; Royo et al., 2019). Among the exceptional features of SH, the homeostasis of the protein Tau is of major interest. Indeed, while Tau hyperphosphorylation causes Alzheimer’s disease in humans (Noble et al., 2013), this hyperphosphorylation is totally reversible in hibernating species and does not cause brain damage (Su et al., 2008). This reversibility, induced by the activity of phosphatases (Liu et al., 2005), prevents brain cellular damage and probably helps to maintain cognitive function, including spatial recognition, social interaction and predator avoidance, at emergence from a hypometabolic state (Figure 1). Nevertheless, it was shown that the activity of these phosphatases is altered during aging (Veeranna et al., 2011, therefore impairing the capacity of Tau dephosphorylation and probably altering the plasticity of the brain of SH with age.

### Alteration of Digestive Capacity

In several SH, the decrease in food quantity and quality associated with the unfavorable season leads to changes in gut size and structure (reviewed in Canale and Henry, 2010) to maintain their energy and protein metabolism (Figure 1). This reversible gut atrophy leads to additional energetic costs to maintain a functional digestive tract through the unfavorable season (Humphries et al., 2003). In addition, the gut microbiota composition is also flexible, and the intestinal microbial diversity changes in response to food composition (Carey et al., 2013; Stevenson et al., 2014; Amato et al., 2015, 2016; Hatton et al., 2017; Kaczmarek et al., 2017; Carmody et al., 2019; Hauffe and Barelli, 2019). The gut microbiota has been extensively associated with energy homeostasis and metabolic control (Wang et al., 2017), particularly in relation to the effects of microbial metabolites on the gut-brain axis (Clemmensen et al., 2017; Cryan et al., 2019). More precisely, the gut microbiota has a major impact on digestive efficiency and on the nutrients that are rendered available for energy homeostasis (Scheithauer et al., 2016; Riedl et al., 2017). The loss of intestinal microbial diversity has been proven to be detrimental to energy balance and mental health (Cryan et al., 2019). Finally, under a hypometabolic state, protein synthesis is inhibited, and it is known that protein deficiency leads to a decrease in tissue restoration capacity (Humphries et al., 2003). The gut remodeling that operates at each seasonal transition may induce a functional mismatch between food availability and digestive efficiency, therefore impairing metabolic control and cognitive function.

### Immunodeficiency

Low body temperature has been linked to reductions in tissue lesions and avoidance of hypoxia during severe systemic inflammation (Liu et al., 2012; Corrigan et al., 2014). Therefore, this dichotomy in host defense might benefit heterotherms during torpor. However, regulated hypometabolism also leads to reduced immune efficiency (Figure 1). For example, in several SH, hypometabolism leads to a decrease in the number of circulating leukocytes, a loss of proliferative capacity of lymphocytes and a decrease in the capacity to induce a cellular response (Bouma et al., 2010). In the case of an infection by a pathogen resistant to cold temperatures, organisms in hypothermia would be immunodeficient (Canale and Henry, 2012). Even if the infection triggers a reactivation of metabolic activity, the immune system could be too late to efficiently cope with the infection. Moreover, depending on its severity, infection will lead to either resistance (through microbicidal mechanisms, for example) or tolerance (through management of collateral damage; Medzhitov et al., 2012; Ganeshan et al., 2019; Steiner and Romanovsky, 2019).

## Conclusion and Perspectives in the Context of Global Warming

In this article, we focused on five potential physiological costs of heterothermy, i.e., oxidative stress, excess fat, cognitive defects, digestive inefficiency, and immunodeficiency, which seem the most relevant. Ecological evidence of these costs is lacking, as the benefits of using heterothermy in a constrained, seasonal environment hide these costs. However, captivity annihilates the ecological benefits of using torpor and promotes the emergence of damage, especially during aging. Therefore, experimental studies in photoentrained heterothermic species should be further considered to assess the costs of physiological remodeling at each key phase of seasonal transition (Figure 1). Other physiological costs may arise, such as the inhibition of mitosis and protein synthesis during torpor and their rapid reactivation at arousal (Humphries et al., 2003) or imperfect phenotype-environment matching during ontogeny that is costly to compensate once growth is terminated (Auld et al., 2010; Dammhahn et al., 2017).

Understanding how heterothermy comes with ecological and physiological costs for seasonal organisms will provide insights into the constraints and physiological processes underlying the reaction norms of phenotypic performances and fitness components to seasonal environmental change (Boyles et al., 2020). This will help us to better understand physiological processes such as circannual biological rhythms and reproductive cycles (Cayetanot et al., 2005). In addition, such studies may shed light on the mechanisms associated with degrading health conditions at old ages (high survival syndrome of hibernators; Turbill et al., 2011) and better prevent physiological disorders such as obesity (Terrien et al., 2017).

Moreover, understanding the costs of heterothermy will allow us to better understand how seasonality influences the evolution of life cycles, life-history traits, such as senescence, and life-history trade-offs. For example, it is important to know whether these costs affect important fitness components, such as growth or young adult survival and fecundity, or affect only old individuals through faster senescence.

The reasoning in the present perspective paper was focused on circannual metabolic changes, but many of these same changes also occur within days (circadian metabolic changes, i.e., peak oxidative stress during phases of catabolism or arousal from torpor, sleep dept, tau phosphorylation of the central nervous system). Hence, the physiological costs that damage the organism over the long term (seasonal cycles) may actually also operate on a daily basis (daily cycles; Melvin and Andrews, 2009).

Finally, given the major ongoing changes and variability in environmental conditions, the benefits of seasonal metabolic changes may decrease (higher winter temperature, increased winter resource availability). If the costs remain unchanged, the cost/benefit imbalance should reduce the net selective advantage of seasonally flexible genotypes, with a reduction in net immediate survival, reproduction, or growth, or an increase in the aging rate. Costs that are negligible compared to benefits in current environments may become crucial in determining future population dynamics in the case of environmental changes. In this respect, opportunistic heterotherms but not SH should be advantaged by environmental changes (Nowack et al., 2017). Indeed, there might exist a mismatch between unpredictable fluctuations in resource availability that do not follow seasonal patterns and the obligatory physiological remodeling undergone by SH, synchronized on variations in the photoperiod. We know that phenological plasticity can be sex- and reproductive phase-dependent and that different timings of hibernation and reproduction are observed in different populations of arctic ground squirrels (Williams et al., 2012, 2017). However, in the case of environmental changes, in which strong SH are unable to plastically adapt, the gap between seasonal and opportunistic heterotherms might increase, which could alter the adaptive value of being seasonally plastic.

## Data Availability Statement

The original contributions presented in the study are included in the article, further inquiries can be directed to the corresponding author.

## Author Contributions

JL, SP, P-YH, and JT contributed to the conception and writing of the manuscript. All authors contributed to the article and approved the submitted version.

## Conflict of Interest

The authors declare that the research was conducted in the absence of any commercial or financial relationships that could be construed as a potential conflict of interest.
